# An Oversampling Method of Unbalanced Data for Mechanical Fault Diagnosis Based on MeanRadius-SMOTE

**DOI:** 10.3390/s22145166

**Published:** 2022-07-10

**Authors:** Feng Duan, Shuai Zhang, Yinze Yan, Zhiqiang Cai

**Affiliations:** School of Mechanical Engineering, Northwestern Polytechnical University, Xi’an 710072, China; fengduan@mail.nwpu.edu.cn (F.D.); yanyinze@mail.nwpu.edu.cn (Y.Y.); caizhiqiang@nwpu.edu.cn (Z.C.)

**Keywords:** mechanical fault diagnosis, unbalanced data set, MeanRadius-SMOTE, minority class

## Abstract

With the development of machine learning, data-driven mechanical fault diagnosis methods have been widely used in the field of PHM. Due to the limitation of the amount of fault data, it is a difficult problem for fault diagnosis to solve the problem of unbalanced data sets. Under unbalanced data sets, faults with little historical data are always difficult to diagnose and lead to economic losses. In order to improve the prediction accuracy under unbalanced data sets, this paper proposes MeanRadius-SMOTE based on the traditional SMOTE oversampling algorithm, which effectively avoids the generation of useless samples and noise samples. This paper validates the effectiveness of the algorithm on three linear unbalanced data sets and four step unbalanced data sets. Experimental results show that MeanRadius-SMOTE outperforms SMOTE and LR-SMOTE in various evaluation indicators, as well as has better robustness against different imbalance rates. In addition, MeanRadius-SMOTE can take into account the prediction accuracy of the overall and minority class, which is of great significance for engineering applications.

## 1. Introduction

With the continuous innovation of technology, industrial equipment has developed rapidly in the direction of large-scale, automated, integrated, and intelligent, such as aircraft engines, steam turbines, wind turbines, centrifuges, etc. In order to meet the requirements of mechanical equipment reliability and precision in the industrial field, PHM (Prognostics and Health Management) was initiated to ensure the stable operation of mechanical equipment and reduce maintenance costs [1,2,3].

With the development of big data in the industrial field, data-driven mechanical fault diagnosis research has received more and more attention [4,5,6]. Mechanical fault diagnosis generally starts by extracting vibration signals from the operation of the equipment, because vibration signals can provide sufficient fault features to reflect the fault status and serve as the input of the prediction model [7,8]. However, due to the low frequency of some faults, the vibration signals of such faults are too small, and the classifier cannot predict them accurately, which is the problem of unbalanced data sets in fault diagnosis. In the multi-classification mechanical fault diagnosis problem, the machine learning classifier emphasizes the accuracy of the overall prediction, which leads to sacrificing the prediction accuracy of the minority class to ensure the prediction of the majority class samples [9]. However, there are infrequent failures in some mechanical equipment, which will lead to huge economic losses once they occur. Therefore, it is necessary to research the problem of unbalanced data sets in mechanical fault diagnosis.

At present, the research on the problem of unbalanced data sets is relatively mature, but this research in the mechanical fault diagnosis field has just begun [10]. Many fault diagnosis techniques rely on reliable and complete data sets, such as multi-sensing fusion techniques [11]. However, since machinery usually operates under normal conditions, it is difficult to collect enough failure data, so that the actual data set lacks completeness [12,13]. The lack of samples with specific labels can lead to data imbalance problems. In recent years, many scholars have begun to pay attention to this problem and have given their own methods [14,15]. Generally, the solution to the problem of unbalanced data sets is mainly divided into data and algorithm aspects, and sometimes they are combined [16].

For the data aspect, scholars mainly use resampling technology to copy, synthesize, delete, and perform other operations on original samples, to adjust the number of samples to reduce the impact of unbalanced data sets. Resampling techniques are divided into oversampling for minority class samples and undersampling for majority class samples. The main idea of oversampling is to increase the number of minority class samples to achieve class balance. The main methods are divided into replicating samples and generating new samples. ROS (Random Oversampling) is to randomly replicate original samples to expand the number of minority class samples, but it may cause the replication of noise samples to affect the quality of the data set [17]. The method of generating new samples derives new samples from one or more original samples, and the new samples can indirectly reflect the features of the minority class. The most classic oversampling is the SMOTE algorithm [18]. The SMOTE algorithm selects the line connecting the two original samples as the range of the new sample and determines a point on the line as the new sample. However, SMOTE still does not avoid the generation of noise samples, and the new samples are easily affected by the distribution of the original samples, which may cause the new samples to deviate from the actual distribution. Later scholars improved SMOTE in terms of noise reduction and generation algorithms, such as Borderline-SMOTE [19], Adasyn [20], LR-SMOTE [21], etc. Undersampling achieves class balance by reducing the number of majority class samples, such as undersampling based on the clustering algorithm and ENN (Edited Nearest Neighbor) [22]. In fact, most of the unbalanced data sets are caused by too few samples in the minority class, so oversampling is the key research in this field [23].

For the algorithm aspect, with the rapid development of machine learning, many classifiers have responded to the problem of unbalanced data sets. On the premise that each sample is equal, the number of samples determines which class the classifier prefers, so setting the weight of the sample, the threshold of the decision boundary, or the objective function of the classifier can strengthen the ability of the classifier to combat unbalanced data sets [24,25]. Adjusting these can make the classifier’s decision boundary less sensitive to the sample size [26]. Moreover, adding a proper regularization term to the objective function can reduce the impact of the imbalance rate on the classifier [27].

There is no universal solution to the problem of unbalanced data sets in mechanical fault diagnosis; although, scholars have tried in various directions. From the perspective of features, extracting more abundant features from vibration signals is beneficial to solving the problem, because the failure can be reflected in the energy of the vibration of the equipment [28]. In addition to features in the time and frequency domains, there are features based on wavelet packet energy and entropy values [29,30], and the fault features are also extracted using a bag-of-visual-word approach from the infrared thermography images [31]. However, the increase of features will undoubtedly increase the workload of feature screening. From the perspective of resampling, scholars use various existing resampling methods to conduct experiments on mechanical equipment [32]. Once there are more failure types or concurrent failures, existing oversampling algorithms may fail. Therefore, analyzing the commonality of mechanical faults and proposing a new oversampling algorithm is the key to solving this problem in the mechanical field [33,34]. From the perspective of the classifier, scholars mainly set the cost matrix, and change the loss function or network structure to make the classifier aware of this imbalance [35]. These classifiers are often only suitable for identifying faults in stationary parts, such as gears or bearings [36].

Although new oversampling algorithms are emerging, there are still the following problems: (1) The solutions are generally only aimed at the prediction of bearing failures or gear failures, so the methods cannot comprehensively diagnose the running state of complete mechanical equipment. (2) Most of the solutions are aimed at the two-category problem, which is obviously not practical. For a simple secondary planetary gear, there are already as many as eight failure types. (3) The new samples are not effective enough that the existing oversampling methods generate. Although the number has reached a balance, it is far from enough in terms of the amount of fault-type information contained in the sample.

In view of the existing problems, this paper improves SMOTE and proposes an oversampling algorithm called MeanRadius-SMOTE, which is specially used to solve the multi-classification problems in mechanical fault diagnosis. MeanRadius-SMOTE can reduce the production of noise samples and add more samples with the ability to affect the decision boundary, and it is easier to inherit the feature information from the original samples. The complexity of the MeanRadius-SMOTE algorithm is not high compared to SMOTE.

The main contributions of this paper are as follows: To solve the problem of multi-classification unbalanced data sets in mechanical fault diagnosis, a new oversampling algorithm, MeanRadius-SMOTE, is proposed. The algorithm takes into account the performance of prediction of overall and minority class, and especially in the minority class, prediction accuracy is greatly improved. In this paper, a large number of comparative experiments are carried out on data sets with various specifications and imbalance rates, and the effectiveness, stability, and robustness of the algorithm are verified.

The rest of this paper is divided into five parts. In Section 2, the SMOTE algorithm and the improved LR-SMOTE algorithm based on SMOTE are introduced. In Section 3, the specific process of the MeanRadius-SMOTE algorithm is introduced in detail. In Section 4, we mainly introduce the source and processing of the data set, as well as the selection of classifiers and evaluation indicators in the experiment. In Section 5, we introduce the experimental process and experimental results. In the following sections, we discuss and summarize the MeanRadius-SMOTE algorithm based on experiments, and we propose future research directions.

## 2. Related Works

Since the machine learning algorithm is greedy in the face of multi-classification problems, the classifier will give priority to ensuring the highest overall accuracy, resulting in an inaccurate prediction of some minority class samples. In the real industrial field, in the face of some faults with low probability but high maintenance cost, operators hope that the model can accurately predict these faults. Therefore, this section introduces the commonly used methods to deal with unbalanced data sets, namely, the traditional SMOTE method and the improved LR-SMOTE method.

### 2.1. SMOTE

The SMOTE algorithm was proposed by Chaw La et al. in 2002 [18], and the algorithm is an improved method based on ROS. In the SMOTE algorithm, new samples are generated based on the original samples, which has a greater probability of obtaining effective features than random oversampling of new samples. The steps of the SMOTE algorithm are as follows:

(1) For each sample *x* in the training set, calculate their Euclidean distance to each minority class sample *x_i_*, and obtain the k nearest neighbors of each minority class sample.
(2) According to the sample imbalance rate, set the sampling ratio N. For *x_i_*, randomly select N samples from its k nearest neighbors, denoted as *x*_h_.
(3) According to Equation (1), build new samples based on *x_i_* and *x*_h_ until the classes are balanced, denoted as *x*_new_.

(1) $$x_{\mathrm{new}}=x_{i}+rand(0,1)*(x_{h}-x_{i})$$

Although the SMOTE algorithm overcomes the overfitting problem of the ROS algorithm, SMOTE still has some problems with noise samples and useless samples. Many scholars have improved SMOTE. For example, Han proposed the Borderline-SMOTE algorithm [19]. The algorithm first classifies the original samples into safe, dangerous, and noise, then uses the dangerous samples to generate new samples. It not only reduces the interference of noise points but also enables new samples to better reflect the features of the data set. However, how to accurately divide the three labels is a more difficult problem for different data sets.

### 2.2. LR-SMOTE

Based on the SMOTE algorithm, Wang proposed the LR-SMOTE algorithm [21]. The algorithm first uses SVM (Support Vector Machine) and K-means to remove the noise samples in the original data set, then changes the generation rules of new samples and considers the center of the samples to generate new samples. The specific steps of the LR-SMOTE algorithm are as follows:

(1) Use SVM to classify the data set, and then for the wrongly classified minority samples use the K-means method to judge and remove the noise samples.
(2) Use K-means to find the center *x_c_* of the minority class sample, calculate the distance *d_i_* from each minority class sample to the center *x_c_*, and calculate the average distance *d*_m_.
(3) For each minority class sample *x_i_*, calculate the ratio *M_i_* of the average distance *d*_m_ and the distance *d_i_*.
(4) According to the number of the same samples in the neighbor samples, set the weight of each minority class sample, and then randomly select a minority class sample *x_i_* and build new samples *x_new_* according to Equation (2).
  (2) $$x_{new}=x_{i}+rand(0,M_{i})*(x_{c}-x_{i})$$
(5) Repeat steps 3 and 4 until the number of samples of the majority class and minority class is balanced.

In the LR-SMOTE algorithm, the new samples are generated based on the functional relationship between the sample center and each sample, rather than any two minority samples. Therefore, the new samples will not deviate from the range of the minority samples, and the features are closer to the original sample. LR-SMOTE provides a good direction for generating rules so that the algorithm determines the distribution of samples according to the sample center. This paper also proposes a new algorithm along this way to solve the unbalanced data sets in the mechanical field. We use the MeanRadius-SMOTE algorithm to experiment on a variety of mechanical failure data sets, and the experimental results show that the MeanRadius-SMOTE algorithm is suitable for solving the problem of unbalanced data sets in the mechanical field.

## 3. Proposed Method

In an oversampling algorithm, new samples at different geometric locations have different improvements in classifier training. In general, the more new samples near the decision boundary, the greater the impact on the classifier. This paper proposes the MeanRadius-SMOTE (MR-SMOTE) algorithm considering the sample center and radius. When using machine learning to predict mechanical failures, we deal with noise samples in advance, so noise reduction is performed in feature preprocessing. Noise reduction is not involved in the MeanRadius-SMOTE, and the noise reduction algorithm will be introduced in the next section.

The MeanRadius-SMOTE algorithm mainly changes the generation rules of the SMOTE algorithm, so that the new samples are more likely to be distributed near the average radius of the minority class samples, and the new samples have a stronger ability to affect the decision boundary of the classifier. In the MeanRadius-SMOTE algorithm, the new sample is determined by k vectors of the sample center to the samples, and the distance between the new sample and the sample center follows a normal distribution. The steps of the MeanRadius-SMOTE algorithm are as follows:

(1) According to each minority class sample, calculate the geometric center, denoted as the sample center *x*_c_ of the minority class sample.
(2) Calculate the Euclidean distance from each minority class sample to the sample center, and then obtain the average distance, denoted as the sample radius *d*_m_ of the minority class.
(3) Randomly select k minority class samples, and then obtain k vectors **v***_i_* from the sample center *x_c_* to the samples. Compute the resultant vector of k vectors.
(4) Use a normal distribution with mean *d*_m_ and variance $\frac{d_{m}}{\theta }$ to determine the distance between the new sample and the sample canter. According to Equation (3), build new samples.
  (3) $$x_{new}=x_{c}+r*\sum_{i=0}^{k}v_{i}\;r∼N(d_{m},\;\frac{d_{m}}{\theta })$$
(5) Repeat steps 3 and 4 until the number of samples of the majority class and minority class is balanced.

**Figure 1:**
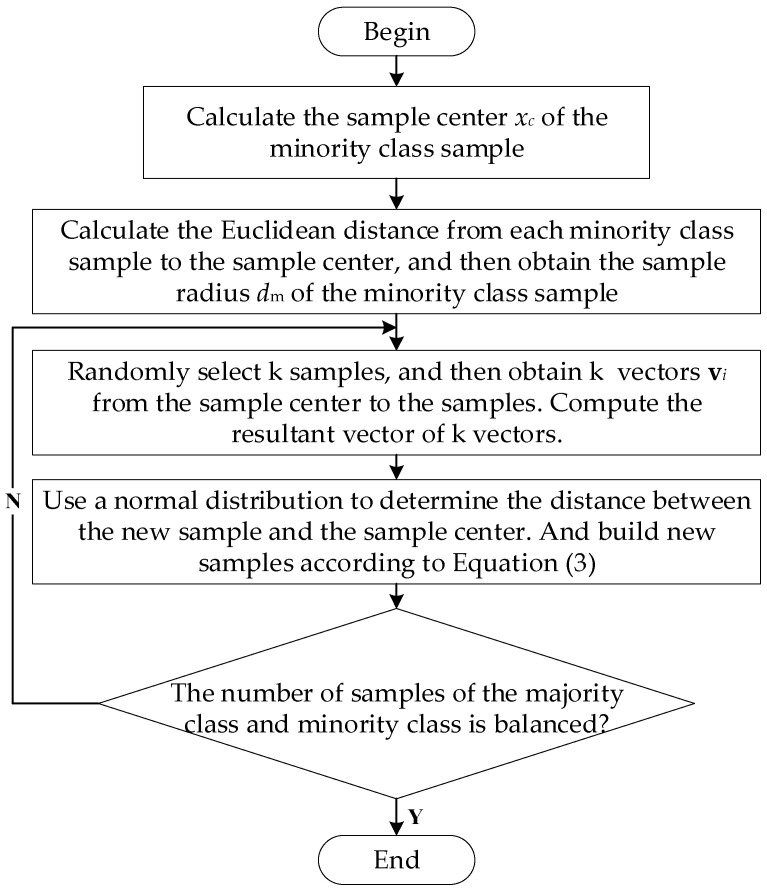
The flow chart of the MeanRadius-SMOTE algorithm.

In order to show the flow of the algorithm more conveniently, we draw the flow chart of the MeanRadius-SMOTE algorithm, as shown in Figure 1.

**Figure 2:**
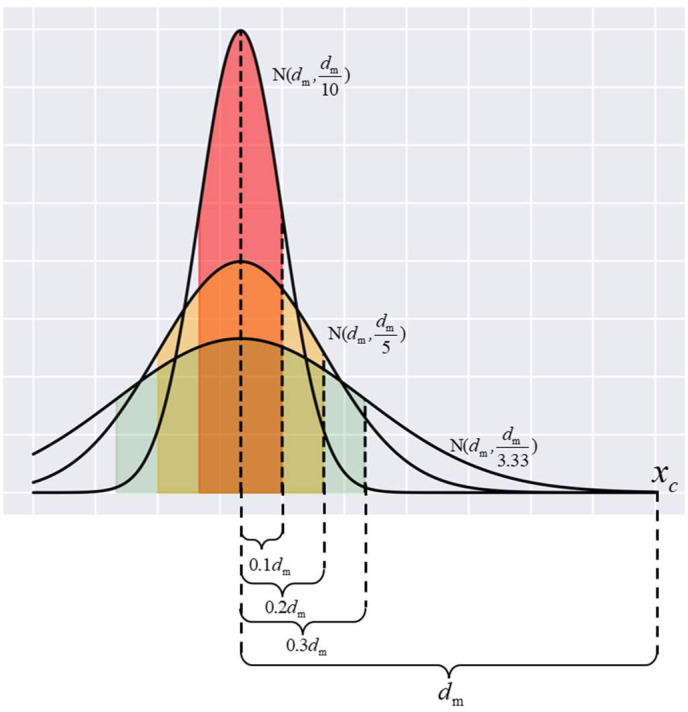
New samples distribution under different θ.

In the MeanRadius-SMOTE algorithm, k and θ are hyperparameters of the algorithm, which are determined according to the number of minority class samples and the imbalance rate. If k is too large, the direction of the new sample relative to the sample center will become meaningless, and θ directly affects the distribution of the new sample. As shown in Figure 2, new samples under different θ are likely to be distributed in colored areas. When θ is too small, the new sample may be far from the sample center. When θ is too large, the new sample is too conservative and cannot balance the number of positive and negative samples near the decision boundary. Therefore, in general, the selection range of parameters k is 2 to 5 and the selection range of parameters θ is 4 to 10.

**Figure 3:**
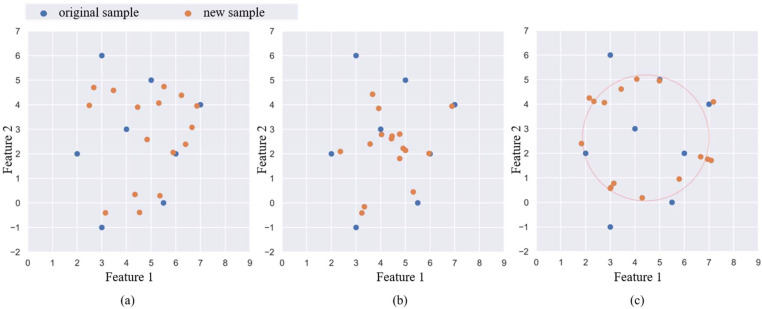
New samples on oversampling algorithms: (**a**) SMOTE, (**b**) LR-SMOTE, (**c**) MeanRadius-SMOTE.

For mechanical equipment, some concurrent faults and the original fault have similar vibration states, and the two types of samples often overlap in distribution. Whether the classifier can find an excellent decision boundary is the key to determining the accuracy of the model. In the MeanRadius-SMOTE algorithm, most of the new samples are concentrated around the sample radius to ensure the validity of the new samples. The new sample is determined by k samples and is related to the sample center, so that the new sample can better inherit the features of the minority class. The geometric positions of new samples generated by different oversampling algorithms have their own characteristics, so we plot the examples of SMOTE, LR-SMOTE, and MeanRadius-SMOTE on two-dimension feature samples, as shown in Figure 3. The information of the two-dimension feature samples is shown in Table 1.

The new samples of SMOTE are more inclined to be generated in locations with a high density of the original samples. Since LR-SMOTE randomly chooses a sample to determine the orientation of the new sample, the new sample is more clustered and radial. In MeanRadius-SMOTE, the orientation of new samples is relatively random, and the new samples are generated around the sample radius.

## 4. Experimental Preparation

### 4.1. Data Set

**Figure 4:**
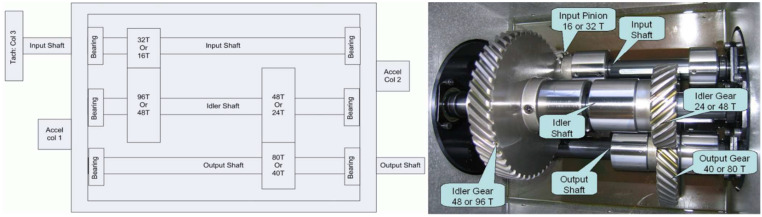
Gearbox used in PHM 2009 challenge data.

Our experimental data set is the 2009 PHM data challenge of gearbox [37]. The data set is a typical industrial gearbox data set, which contains 3 shafts, 4 gears, and 6 bearings, and its experimental bench is shown in Figure 4. The data set tests two sets of gears: spur gear and helical gear. The spur gear data set contains 8 health states, and the helical gear data set contains 6 health states. The data set consists of two channels of accelerometer signals and one channel of tachometer signals. The sampling frequency is 66.67 kHz, and the tachometer signals are collected at 10 pulses per revolution. There are five types of shaft speeds: 30 Hz, 35 Hz, 40 Hz, 45 Hz, and 50 Hz, with high and low loads. In the experiment, we chose the low load spur gear operating data at 30 Hz, and we used the vibration data of the two acceleration channels for feature extraction, The 8 health states of spur gears are as follows in Table 2.

Mechanical equipment frequently fails in the harsh environment of high temperature and high pressure due to concurrent failures composed of multiple single failures [38]. In the PHM dataset, there are many types of concurrent failures, such as labels 4 to 8. They are all combinations of different types of failures in gears and bearings.

For the vibration signal, we sampled the data set using a sliding window with a stride of 100 and a width of 1000. Then we extracted time–frequency domain features for each vibration signal sample and add labels [39]. The formula of 23 features is shown in Table 3.

In the experiment, we used the K-nearest neighbor algorithm to denoise the data set. If the five nearest samples around a sample are not of this class, we consider it to be a noise sample and delete it. After the above preprocessing, we obtained 2656 samples per label, of which 1000 samples per label were taken as the test set. Additional samples were used to construct unbalanced data sets.

### 4.2. Classifiers

In order to comprehensively evaluate the oversampling algorithm, we chose different classifiers to build the experimental model, which excludes the influence of the classifier and verifies the generality of the oversampling algorithm. Through experiments in a large number of mechanical fault diagnoses, the SVM classifier generally has a good training effect, so we chose SVM to establish a classification model. With the continuous development of the decision tree algorithm, the ensemble learning model is also favored by scholars because of its excellent generalization ability. Therefore, we chose RF (Random Forest) representing bagging ensemble mode, and GBDT (Gradient Boosting Decision Tree) representing boosting ensemble mode for experiments.

### 4.3. Evaluation Indicators

Traditional evaluation indicators can well evaluate the performance of the model in the two-category problem. However, in the multi-classification problem, due to the partiality of the classifier, these indicators cannot comprehensively evaluate the model on unbalanced data sets. The expectation of the oversampling algorithm in this paper is to improve the prediction performance of the minority class without losing the overall prediction accuracy of the classifier. Therefore, we will use the traditional evaluation indicators and the prediction indicator of the minority class to evaluate the prediction model. For class i samples, we define the prediction results as follows, as shown in Table 4:

We choose the following four evaluation indicators:

(1) Accuracy (Acc): The Acc value is the ratio of the number of correctly predicted samples to the total number of samples. The calculation method is as shown in Equation (4):
  (4) $$\mathrm{Acc}=\frac{\sum_{n}{\mathrm{TP}}_{i}+{\mathrm{FN}}_{i}}{\sum_{n}{\mathrm{FP}}_{i}+{\mathrm{TN}}_{i}+{\mathrm{TP}}_{i}+{\mathrm{FN}}_{i}}$$
  The Acc value evaluates the overall prediction, but in the case of unbalanced data sets, it is not a good indicator to measure the results.
(2) Macro-Precision (Mac-P): The calculation method of the Precision value for class *i* samples is as shown in Equation (5):
  (5) $${\mathrm{Precision}}_{i}=\frac{{\mathrm{TP}}_{i}}{{\mathrm{TP}}_{i}+{\mathrm{FP}}_{i}}$$
  In the multi-classification problem, the Precision value is divided into Macro and Micro methods. Micro-Precision focuses more on types of samples with a large number of samples, so it is more susceptible to the majority class. However, Mac-P will treat each type of sample equally, so it can better describe the model’s ability to deal with unbalanced data sets. The calculation method is as shown in Equation (6):
  (6) $$\mathrm{Mac}-P=\frac{\sum_{n}{\mathrm{Precision}}_{i}}{n}$$
(3) Macro-F1 (Mac-F1): It is contradictory to improve the Precision value and Recall value at the same time. The F1 value is a balance point with high Precision value and high Recall value, and its calculation method is as shown in Equation (7):
  (7) $${F1}_{i}=\frac{2*{\mathrm{Precision}}_{i}*{\mathrm{Recall}}_{i}}{{\mathrm{Precision}}_{i}+{\mathrm{Recall}}_{i}}$$
  In the multi-classification problem, The F1 value also has Macro and Micro methods such as the Precision value. This paper selects Mac-F1, which can better take into account the minority class. The calculation method is as shown in Equation (8):
  (8) $$\mathrm{Mac}-F1=\frac{\sum_{n}{F1}_{i}}{n}$$
(4) Precision-Minority (Pre*_small_*): In order to pay more attention to the prediction effect of the model on the minority class samples after oversampling algorithms, we will calculate the Precision value of the minority class as an indicator, and its calculation method is as shown in Equation (9):

(9) $${\mathrm{Pre}}_{small}=\frac{{\mathrm{TP}}_{small}}{{\mathrm{FP}}_{small}+{\mathrm{TP}}_{small}}$$

## 5. Experimental Design and Results

**Figure 5:**
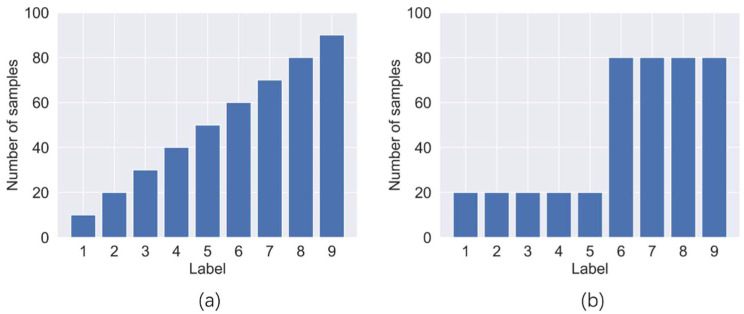
Two imbalance forms: (**a**) linear imbalance, (**b**) step imbalance.

In this paper, we will design unbalanced data sets of various sizes for experiments. According to the distribution of sample data volume within each class, unbalanced data sets can be divided into two forms, linear imbalance and step imbalance. The distribution of sample data volume for the two forms is as shown in Figure 5.

In this paper, we design three linear unbalanced data sets and four step unbalanced data sets. In order to reduce the interference of the class on the Pre*_small_* in different experiments, we set the number of samples for labels 4 to 50 as the smallest minority class. We set the normal label as the large sample class, and the imbalance rate is designed to be 30, 20, and 15, through which the number of other labels can be determined. The details of the seven unbalanced data sets are shown in Table 5. For line-1 to 3, their imbalance rates are not the same. Moreover, the label linear order is shuffled. For stage-1 to 4, there are differences in the imbalance rate and the ratio of minority class and majority class labels.

In the experiment, we will use the SMOTE, LR-SMOTE, and MeanRadius-SMOTE to oversample the seven unbalanced data sets, so that each class label becomes balanced. Then, we conduct experiments on the original data set and the three processed data sets on SVM, RF, and GBDT classifiers. In order to eliminate the training bias caused by random data, all experiments were performed with 5-fold cross-validation and repeated 10 times to obtain the average number of indicators.

The experimental results of Acc, Mac-P, and Mac-F1 on the linear unbalanced data sets and step unbalanced data sets are shown in Table 6 and Table 7, where the values with bold mean the largest value in four compared models.

From Table 6, it can be found that the oversampling algorithm can effectively improve Acc, Mac-P, and Mac-F1, and MeanRadius-SMOTE is the best in most cases. In some experiments, SMOTE performs better than MeanRadius-SMOTE, but the gap between them is very small. However, in the SVM experiment, MeanRadius-SMOTE improves the three indicators much better than SMOTE and LR-SMOTE.

From Table 7, since there are more minority classes in the step unbalanced data sets, the three indicators are all lower in the experiments without oversampling, and are more affected by the imbalance rate. The SVM classifier combined with any oversampling algorithm is better than the ensemble learning classifier, and there are obvious gaps in the three indicators. On the step unbalanced data sets, MeanRadius-SMOTE outperforms SMOTE and LR-SMOTE in all cases, and the gap is especially significant on the SVM classifier.

By analyzing Acc, Mac-P, and Mac-F1, all oversampling algorithms can effectively improve the overall prediction performance of the classifier on both forms of unbalanced data sets, and the MeanRadius-SMOTE algorithm proposed in this paper has the most obvious effect. We still need to focus on the prediction performance of the algorithm on the minority class; the experimental results of Pre*_small_* are shown in Table 8, where the values with bold mean the largest value in four compared models.

From Table 8, Pre*_small_* does not even exceed five in the None experiments. SMOTE and LR-SMOTE only improved Pre*_small_* by around five in most experiments. However, MeanRadius-SMOTE can help the classifier to more accurately predict the minority class, improving Pre*_small_* by around six or seven. In addition, MeanRadius-SMOTE is more stable in experiments with different imbalance rates, and does not fluctuate greatly like SMOTE and LR-SMOTE.

**Figure 6:**
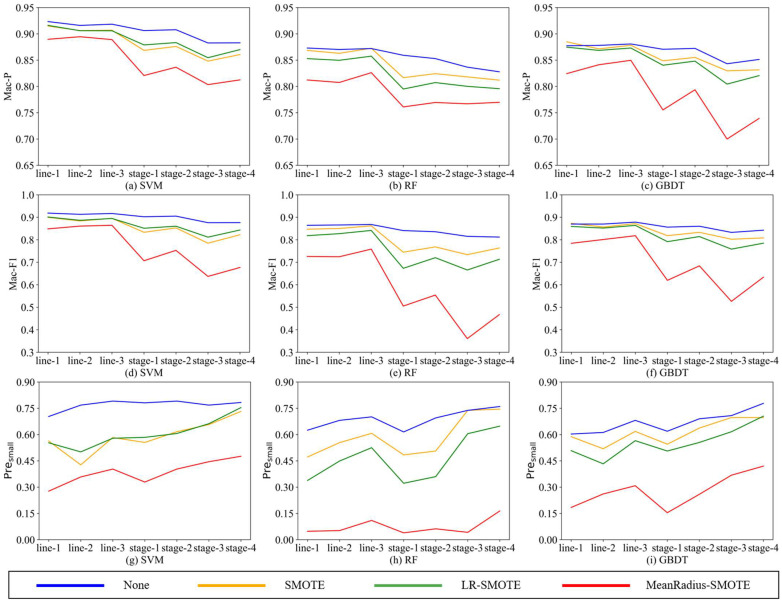
The line charts of Mac-P, Mac-F1, and Pre*_small_*_._

To better compare the effects of SMOTE, LR-SMOTE, and MeanRadius-SMOTE, we draw the line charts of Mac-P, Mac-F1, and Pre*_small_*, as shown in Figure 6. Since the data of Acc and Mac-F1 are close and their trend is basically the same, we only choose Mac-F1 to draw the line chart.

According to Figure 6, the following conclusions can be drawn:

(1) Since these seven unbalanced data sets are homologous, the better the oversampling algorithm, the closer the indicators should be. Comparing the nine charts, all indicators are relatively stable in the MeanRadius-SMOTE experiment, which is less affected by the imbalance rate and data set form, and this stabilization is more obvious in the SVM classifier. This shows that MeanRadius-SMOTE has good robustness.
(2) Analyzing the three charts—Figure 6a,d,g, in the seven data sets, MeanRadius-SMOTE on the SVM classifier can not only ensure that the overall prediction indicators reach about 0.9 but also ensure that Pre*_small_* is relatively high, about 0.75.
(3) Comparing the three charts—Figure 6g–i, the SVM experiment can achieve a higher Pre*_small_*, and in most experiments, Pre*_small_* is greatly affected by the data sets, especially in the RF experiments. However, only in the model composed of MeanRadius-SMOTE and SVM do we obtain a very flat line, which shows that this model has good robustness and accuracy in predicting the minority class.
(4) Comparing the three charts—Figure 6a–c, for SMOTE and LR-SMOTE, LR-SMOTE performs better than SMOTE on SVM, while it is the opposite on RF and GBDT. In addition, SMOTE even outperforms MeanRadius-SMOTE in some GBDT experiments. LR-SMOTE is also an oversampling algorithm for binary classification problems, which is more suitable for a classifier that is essentially a binary classification algorithm-SVM. Therefore, it can be inferred that MeanRadius-SMOTE is also more suitable for SVM classifiers.

In summary, MeanRadius-SMOTE shows excellent performance in all experiments, which can take into account the prediction performance of the overall and minority class. In individual experiments, SMOTE is slightly higher than MeanRadius-SMOTE in Acc, Mac-P, and Mac-F1, but lower than MeanRadius-SMOTE in Pre*_small_*. We can think that this is the result of sacrificing the prediction performance of the minority class. Therefore, it can still be considered that MeanRadius-SMOTE is better than SMOTE and LR-SMOTE. Furthermore, the model composed of MeanRadius-SMOTE and SVM can improve prediction accuracy and stability.

## 6. Conclusions and Outlook

Mechanical fault diagnosis has always been a key issue in the PHM. Since the development of machine learning, although mechanical fault diagnosis has been solved by many effective methods, fault diagnosis under unbalanced data sets has always been a stubborn problem. The oversampling algorithm is currently recognized as an effective means to solve the problem of unbalanced data sets. The traditional oversampling algorithm is not only affected by the sample distribution, but also easily generates noise samples, which makes the decision boundary blurred. These drawbacks are not conducive to the classifier making predictions.

Based on the SMOTE, this paper proposes the new algorithm, MeanRadius-SMOTE, combining the sample center and radius. MeanRadius-SMOTE effectively avoids useless samples and noise samples in the process of generating new samples. In this paper, we conduct comparative experiments for SMOTE, LR-SMOTE, and MeanRadius-SMOTE algorithms and use SVM, RF, and GBDT classifiers on three linear unbalanced data sets and four step unbalanced data sets. Experimental results show that the MeanRadius-SMOTE algorithm can effectively balance data classes and improve the prediction performance of machine learning classifiers. From the perspective of various indicators, the MeanRadius-SMOTE algorithm is better than SMOTE and LR-SMOTE, and has better robustness. In the problem of unbalanced data sets, MeanRadius-SMOTE can more accurately predict the minority class without sacrificing the prediction performance of other classes, which is of great significance for mechanical fault diagnosis, and the combined model of MeanRadius-SMOTE and SVM is proved to be much better than other models.

Although this paper proves on PHM09 challenge data that MeanRadius-SMOTE has a good ability to deal with unbalanced data sets, considering the actual situation, future research can be carried out from the following aspects:

(1) In this paper, in order to ensure that the experiment is carried out under a variety of unbalanced data sets, we use artificial unbalanced data sets in experiments. In future research, we will collect the failure unbalanced data sets of actual mechanical equipment to continue the verification experiment.
(2) When constructing the data set in this paper, we only extracted the time–frequency domain features from the vibration signal. Currently, there are more methods to extract features from vibration signals, such as convolutional neural networks, wavelet packet decomposition, etc. Training sets composed of different types of features may have an impact on the performance of MeanRadius-SMOTE.

## Figures and Tables

**Table 1:** The information of the two-dimension feature samples.

	Sample 1	Sample 2	Sample 3	Sample 4	Sample 5	Sample 6	Sample 7	Sample 8
Feature 1	3	4	6	7	5	2	3	5.5
Feature 2	6	3	2	4	5	2	−1	0

**Table 2:** A brief description of the faults.

Label	Description
Label 1	Good
Label 2	Gear chipped and eccentric
Label 3	Gear eccentric
Label 4	Gear eccentric and broken, bearing ball fault
Label 5	Gear chipped and eccentric and broken, bearing inner and ball and outer fault
Label 6	Gear broken, bearing inner and ball and outer fault, shaft imbalance
Label 7	Bearing inner fault, shaft keyway sheared
Label 8	Bearing ball and outer fault, shaft imbalance

**Table 3:** The time–frequency domain features.

Time-Domain Feature		Frequency-Domain Feature	
$F_{1}=\frac{\sum_{n=1}^{N}x(n)}{N}$	$F_{7}=\frac{\sum_{n=1}^{N}{\left(x(n)-F_{1}\right)}^{4}}{(N-1)F_{2}{}^{4}}$	$F_{12}=\frac{\sum_{k=1}^{K}s(k)}{K}$	$F_{18}=\sqrt{\frac{\sum_{k=1}^{K}f_{k}{}^{2}s(k)}{\sum_{k=1}^{K}s(k)}}$
$F_{2}=\sqrt{\frac{\sum_{n=1}^{N}{\left(x(n)-F_{1}\right)}^{2}}{N-1}}$	$F_{8}=\frac{F_{5}}{F_{4}}$	$F_{13}=\frac{\sum_{k=1}^{K}{\left(s(k)-F_{12}\right)}^{2}}{K-1}$	$F_{19}=\sqrt{\frac{\sum_{k=1}^{K}f_{k}{}^{4}s(k)}{\sum_{k=1}^{K}f_{k}{}^{2}s(k)}}$
$F_{3}={\left(\frac{\sum_{n=1}^{N}\sqrt{x(n)}}{N}\right)}^{2}$	$F_{9}=\frac{F_{5}}{F_{3}}$	$F_{14}=\frac{\sum_{k=1}^{K}{\left(s(k)-F_{12}\right)}^{3}}{K(\sqrt{F_{13}}{)}^{3}}$	$F_{20}=\frac{\sum_{k=1}^{K}f_{k}{}^{2}s(k)}{\sqrt{\sum_{k=1}^{K}s(k)\sum_{k=1}^{K}f_{k}{}^{4}s(k)}}$
$F_{4}=\sqrt{\frac{\sum_{n=1}^{N}{\left(x(n)\right)}^{2}}{N}}$	$F_{10}=\frac{F_{4}}{\frac{1}{N}\sum_{n=1}^{N}\left|x(n)\right|}$	$F_{15}=\frac{\sum_{k=1}^{K}{\left(s(k)-F_{12}\right)}^{4}}{KF_{13}{}^{2}}$	$F_{21}=\frac{F_{17}}{F_{16}}$
$F_{5}=max\left|x(n)\right|$	$F_{11}=\frac{F_{5}}{\frac{1}{N}\sum_{n=1}^{N}\left|x(n)\right|}$	$F_{16}=\frac{\sum_{k=1}^{K}f_{k}s(k)}{\sum_{k=1}^{K}s(k)}$	$F_{22}=\frac{\sum_{k=1}^{K}{\left(s(k)-F_{16}\right)}^{3}s(k)}{KF_{17}{}^{3}}$
$F_{6}=\frac{\sum_{n=1}^{N}{\left(x(n)-F_{1}\right)}^{3}}{(N-1)F_{2}{}^{3}}$		$F_{17}=\frac{\sum_{k=1}^{K}{\left(f_{k}-F_{16}\right)}^{2}s(k)}{K}$	$F_{23}=\frac{\sum_{k=1}^{K}{\left(s(k)-F_{16}\right)}^{0.5}s(k)}{K\sqrt{F_{17}}}$
where *x(n)* is a signal series for *n* = 1 − N, and N is the number of data points.		where *s(k)* is a signal series for *k* = 1 − K, and K is the number of spectrum lines; *f_k_* is the frequency value of the kth spectrum line.	

**Table 4:** Predicting results for class *i* samples.

	Positive Prediction	Negative Prediction
Positive class	TP*_i_*	FN*_i_*
Negative class	FP*_i_*	TN*_i_*

**Table 5:** Unbalanced data sets description.

Imbalance Forms	Name	Number of Samples								Imbalance Rate
		Label 1	Label 2	Label 3	Label 4	Label 5	Label 6	Label 7	Label 8	
linear	line-1	1500	465	258	50	672	879	1293	1086	30
	line-2	1000	864	592	50	728	321	185	457	20
	line-3	750	550	450	50	150	350	650	250	15
step	stage-1	1500	50	1500	50	1500	1500	1500	50	30
	stage-2	750	50	750	50	750	750	750	50	15
	stage-3	1500	50	1500	50	50	50	50	1500	30
	stage-4	750	50	750	50	50	50	50	750	15

**Table 6:** Experimental results of the linear unbalanced data set.

Data Set	Methods	SVM			RF			GBDT		
		Acc	Mac-P	Mac-F1	Acc	Mac-P	Mac-F1	Acc	Mac-P	Mac-F1
line-1	None	0.8675	0.8896	0.8484	0.7726	0.8122	0.7256	0.8126	0.8243	0.7842
	SMOTE	0.9045	0.9148	0.8997	0.8555	0.8685	0.8471	0.8774	0.8849	0.8731
	LR-SMOTE	0.9065	0.9161	0.9012	0.8339	0.8528	0.8182	0.8662	0.8746	0.8591
	MR-SMOTE	0.9206	0.9233	0.9186	0.8678	0.8730	0.8643	0.8739	0.8773	0.8698
line-2	None	0.8733	0.8945	0.8607	0.7668	0.8075	0.7243	0.8209	0.8412	0.8011
	SMOTE	0.8891	0.9062	0.8836	0.8548	0.8629	0.8501	0.8626	0.8713	0.8566
	LR-SMOTE	0.8923	0.9059	0.8865	0.8354	0.8497	0.8271	0.8588	0.8685	0.852
	MR-SMOTE	0.9139	0.9160	0.9131	0.8675	0.8702	0.8657	0.8733	0.8780	0.8698
line-3	None	0.8754	0.8890	0.8644	0.792	0.8261	0.7583	0.8344	0.8496	0.818
	SMOTE	0.8995	0.9069	0.8954	0.8646	0.8726	0.8618	0.8748	0.8782	0.8716
	LR-SMOTE	0.8988	0.9058	0.8947	0.8464	0.8575	0.8415	0.8683	0.8730	0.8639
	MR-SMOTE	0.9175	0.9183	0.9168	0.8691	0.8720	0.8679	0.8803	0.8807	0.8784

**Table 7:** Experimental results of the step unbalanced data set.

Data Set	Methods	SVM			RF			GBDT		
		Acc	Mac-P	Mac-F1	Acc	Mac-P	Mac-F1	Acc	Mac-P	Mac-F1
Stage-1	None	0.7403	0.8207	0.7066	0.6144	0.7610	0.5051	0.6793	0.7553	0.6194
	SMOTE	0.8418	0.8685	0.8332	0.7614	0.8166	0.7447	0.8250	0.8487	0.8182
	LR-SMOTE	0.8566	0.8789	0.8512	0.7103	0.7950	0.6729	0.8021	0.8403	0.7915
	MR-SMOTE	0.9039	0.9062	0.9023	0.844	0.8592	0.8408	0.8596	0.8706	0.8561
Stage-2	None	0.7746	0.8365	0.7528	0.6398	0.7694	0.5538	0.7193	0.7936	0.6838
	SMOTE	0.8575	0.8760	0.8525	0.7790	0.8242	0.7682	0.8368	0.8551	0.8330
	LR-SMOTE	0.8649	0.8833	0.8602	0.7429	0.8073	0.7202	0.8205	0.8481	0.8142
	MR-SMOTE	0.9064	0.9078	0.9051	0.838	0.8529	0.8357	0.8621	0.8723	0.8601
Stage-3	None	0.6465	0.8034	0.6369	0.4534	0.7669	0.3607	0.5651	0.6999	0.5259
	SMOTE	0.7828	0.8481	0.7847	0.7390	0.8181	0.7336	0.8048	0.8297	0.8022
	LR-SMOTE	0.8118	0.8546	0.8116	0.6766	0.8001	0.6654	0.7641	0.8044	0.7583
	MR-SMOTE	0.8771	0.8826	0.8759	0.8163	0.8366	0.8151	0.8351	0.8431	0.8327
Stage-4	None	0.6823	0.8124	0.6767	0.5186	0.7697	0.4671	0.6491	0.7391	0.6334
	SMOTE	0.8221	0.8606	0.8223	0.767	0.8119	0.7634	0.8098	0.8315	0.8082
	LR-SMOTE	0.8440	0.8700	0.8434	0.7186	0.7957	0.7131	0.7871	0.8205	0.7848
	MR-SMOTE	0.8766	0.8829	0.8762	0.8135	0.8278	0.8119	0.8436	0.8513	0.8425

**Table 8:** Pre*_small_* on the data sets.

Data Set	Methods	SVM Pre*_small_*	RF Pre*_small_*	GBDT Pre*_small_*	Data Set	Methods	SVM Pre*_small_*	RF Pre*_small_*	GBDT Pre*_small_*
line-1	None	0.277	0.048	0.184	stage-1	None	0.329	0.039	0.154
	SMOTE	0.563	0.472	0.588		SMOTE	0.555	0.484	0.545
	LR-SMOTE	0.553	0.338	0.508		LR-SMOTE	0.584	0.322	0.506
	MR-SMOTE	0.703	0.625	0.603		MR-SMOTE	0.781	0.615	0.619
line-2	None	0.358	0.052	0.261	stage-2	None	0.403	0.062	0.259
	SMOTE	0.427	0.554	0.519		SMOTE	0.616	0.506	0.637
	LR-SMOTE	0.501	0.449	0.433		LR-SMOTE	0.606	0.36	0.555
	MR-SMOTE	0.768	0.681	0.612		MR-SMOTE	0.791	0.695	0.690
line-3	None	0.403	0.110	0.308	stage-3	None	0.445	0.042	0.368
	SMOTE	0.583	0.607	0.618		SMOTE	0.657	0.738	0.697
	LR-SMOTE	0.579	0.525	0.565		LR-SMOTE	0.662	0.605	0.616
	MR-SMOTE	0.791	0.701	0.681		MR-SMOTE	0.768	0.738	0.708
					stage-4	None	0.476	0.164	0.42
						SMOTE	0.732	0.745	0.697
						LR-SMOTE	0.754	0.648	0.705
						MR-SMOTE	0.783	0.760	0.778

## Data Availability

The data in this article are the 2009 PHM data challenge of the gearbox.
